# A Label-Free Photoluminescence Genosensor Using Nanostructured Magnesium Oxide for Cholera Detection

**DOI:** 10.1038/srep17384

**Published:** 2015-11-27

**Authors:** Manoj Kumar Patel, Md. Azahar Ali, Sadagopan Krishnan, Ved Varun Agrawal, AbdulAziz A. Al Kheraif, H. Fouad, Z.A. Ansari, S. G. Ansari, Bansi D. Malhotra

**Affiliations:** 1 Biomedical Instrumentation Section, CSIR-National Physical Laboratory, Dr. K. S. Krishnan Marg, New Delhi 110012, India; 2 Centre for Interdisciplinary Research in Basic Sciences, Jamia Millia Islamia, New Delhi 110025, India; 3 Department of Chemistry, College of Arts and Sciences, Oklahoma State University, Stillwater, Oklahoma 74078, United States of America; 4 Department of Electrical and Computer Engineering, Iowa State University, Ames, IA 50011, United States of America; 5 Dental Biomaterials Research Chair, Dental Health Department, College of Applied Medical Science, King Saud University, Riyadh, 11437 Saudi Arabia; 6 Biomedical Engineering Department, Faculty of Engineering, Helwan University, 11792, Egypt; 7 Department of Biotechnology, Delhi Technological University, Shahabad Daulatpur, Main Bawana Road, Delhi 110042, India

## Abstract

Nanomaterial-based photoluminescence (PL) diagnostic devices offer fast and highly sensitive detection of pesticides, DNA, and toxic agents. Here we report a label-free PL genosensor for sensitive detection of *Vibrio cholerae* that is based on a DNA hybridization strategy utilizing nanostructured magnesium oxide (nMgO; size >30 nm) particles. The morphology and size of the synthesized nMgO were determined by transmission electron microscopic (TEM) studies. The probe DNA (pDNA) was conjugated with nMgO and characterized by X-ray photoelectron and Fourier transform infrared spectroscopic techniques. The target complementary genomic DNA (cDNA) isolated from clinical samples of *V. cholerae* was subjected to DNA hybridization studies using the pDNA-nMgO complex and detection of the cDNA was accomplished by measuring changes in PL intensity. The PL peak intensity measured at 700 nm (red emission) increases with the increase in cDNA concentration. A linear range of response in the developed PL genosensor was observed from 100 to 500 ng/μL with a sensitivity of 1.306 emi/ng, detection limit of 3.133 ng/μL and a regression coefficient (R^2^) of 0.987. These results show that this ultrasensitive PL genosensor has the potential for applications in the clinical diagnosis of cholera.

Nanostructured materials are useful building blocks of photoluminescence (PL)-based nano-electronic devices for investigating immunocytochemistry, immunohistochemistry, and protein-protein and DNA-DNA interactions^1^,^2^. For increased PL sensing efficiency, photo-stable nanoparticles (NPs) can be used as sensing nano-probes and energy donors to enable luminescence resonance energy transfer^3^. PL spectroscopy is a powerful optical method for probing electronic structure of desired materials. Non-destructive and contactless PL spectroscopic tools can be used to detect ultrasensitive biomolecules by combining with high intensity luminescent NPs^4^. This technique has the potential to identify minute concentrations of specific impurities that can strongly affect material quality and device performance. Biomolecules conjugated to luminophore-doped silica NPs prepared using water-in-oil micro emulsion method have been explored as photo-stable biomarkers for identification of leukaemia cells^5^. Dye-doped photo-luminescent gold NPs synthesized sonochemically have recently been reported for DNA biosensing^6^. Dual luminophores consisting of entrapped NPs can be utilized for multiplexed signalling in bioanalysis, as NPs may facilitate high signal amplification, excellent photo-stability, and surface bioconjugation^7^. However, unlabeled nanoparticle-based sensing probes have not yet been explored for detection of biomolecules.

Due to high Q-factor, quantum yield, and tunable size and shape properties, nanostructured metal oxides (nMOx) have recently become popular for fabrication of optical diagnostic devices^8^. Besides this, nMOx also have applications in solid state lighting, biomedical labelling, imaging, photodynamic activation, and radiation detection^9^,^10^,^11^,^12^. MgO is widely used as a refractory material, sorbent, catalyst, and catalytic support in catalysis. The particular lattice structure of MgO is responsible for its luminescent properties, which can be used in sensor development^13^. The excellent PL property of nanostructured magnesium oxide (nMgO) with a wide band gap (7.8 eV) can be exploited for the development of PL-based biosensing devices^10^,^14^. MgO has a cubic face-centred Bravais lattice in which anions (O^2–^) and cations (Mg^2+^) are located at octahedral sites with ionic radii of 1.26 and 0.86 Å, respectively. The emission peak at 450 nm in the PL of nMgO can be attributed to the relaxation of polarization defects formed due to strained sites attached to oxygen vacancies. The intrinsic defects observed in nMgO (i.e., oxygen or magnesium vacancies) may result in interesting optical and electron emission properties^15^. Oxygen vacancies such as neutral F centers and positive F^+^ centers are known to have one and two electrons, respectively, that may significantly contribute to PL characteristics of the nMgO. The nature of these F centers in nMgO depends on the synthesis method and doping procedure used. Higher concentrations of these F centers may lead to aggregation or formation of dimeric forms such as FF, FF^+^, and F^+^F^+^^14^.

The PL in thin film of MgO nanocrystals and effect of controlling the size of crystals has recently been investigated^16^,^17^. However, PL property of MgO nanocrystals has not yet been explored for quantification of DNA hybridization. In this context, nMgO can perhaps be used for the development of a photoluminescence based label-free genosensor to investigate DNA hybridization. In addition, the high isoelectric point (IEP, ~12.0) of nMgO may allow strong electrostatic interactions with low IEP molecules such as DNA (IEP, ~5.0), RNA and proteins.

Cholera is water borne infectious disease and the main cause of this disease is polluted water. Highly virulent strains of *V. cholerae* serogroups O1 and O139 are responsible for the infection worldwide^18^. The pathogenesis of cholera is associated with the production of an exotoxin called cholera toxin (CT). Cholera is a serious communicable disease, and it may lead to death if untreated at an early stage^19^. Haddour *et al.* developed a photo-electrochemical immunosensor using a photosensitive biotinylated polypyrrole film for quantification of anti-cholera toxin antibody in the concentration range of 0 to 200 μg/mL^20^. Several research groups have explored the fabrication of low cost and sensitive clinical devices for monitoring cholera based on electrochemical and optical techniques^21^,^22^. However, there is a need for a pathogenic genosensor with improved characteristics^23^.

Here we describe a label-free, sensitive, and stable PL based genosensor that uses chemically synthesized nMgO for *V. cholerae* detection. This nMgO was characterized using X-ray diffraction (XRD), high resolution transmission electron microscopy (HR-TEM), X-ray photoelectron spectroscopy (XPS), and Fourier transform infrared (FT-IR) spectroscopic techniques. Figure 1 schematically shows the construction of the label-free optical PL genosensor.

## Results

Figure 2(A) shows the XRD peaks observed at 2θ values of 36.86°, 42.82°, and 62.17° corresponding to the (111), (200), and (220) planes of standard MgO [JCPDS No. 89-7746]. The observed broadness of the XRD peaks arising from the dominant (200) and (220) planes confirms crystalline nature of the nMgO. The high peak intensity of the plane (200) with full width at the half maximum of 0.98 radians implies that the majority of the grains are oriented along the (200) direction. The average crystallite size (d_200_) of the nMgO is estimated as ~16 nm based on the Scherrer equation for the dominant (200) plane.

The geometrical and morphological observations (e.g., size, shape, and crystallinity) of the synthesized nMgO were carried using high resolution TEM (HRTEM). Image shows that nMgO NPs are randomly shaped while some are hexagonal in shape. The average size of nMgO NPs is <30 nm as estimated from the Fig. 2B. A high resolution image of nMgO shows a mixture of regular and hexagonal geometries (inset, Fig. 2B). The asymmetric growth of nMgO occurs along the (200) crystalline plane^24^, which is in good agreement with results of the XRD studies (Fig. 2A). The lattice fringes of nMgO NPs are shown in Fig. 2C. The lattice spacing is estimated to be 0.21 nm for the (200) plane. Figure 2D shows selected area electron diffraction (SAED) pattern of the nMgO where various planes, such as (111), (200), (220), (311), and (222), of nMgO which are analogus to that of XRD studies.

Figure 3(A) shows the FT-IR spectra obtained before and after immobilization of the probe DNA (pDNA) on the surface of nMgO. The vibrational bands observed at ~445 and 670 cm^–1^ correspond to the Mg-O (Fig. 3A(i)) stretching in the finger print region. After immobilization of the pDNA, new vibrational bands are observed between 800 and 1200 cm^–1^ which appear due to DNA bases. These observations confirms the immobilization of pDNA onto nMgO (Fig. 3A(ii)).

Figure 3(B) shows XPS survey spectrum of nMgO deposited onto indium tin oxide (ITO) glass substrate. The spectrum depicts presence of the oxygen 1s (O*1s*), nitrogen 1s (N*1s*), carbon 1s (C*1s*), and magnesium 2p (Mg*2p*) peaks. The oxygen (O*1s*) peaks were deconvoluted into characteristic components using the Shirley type baseline and Lorentzian-Doniac-Sunsic curves with the Gaussian profile. Figure 3(C) shows the characteristic oxygen (O) *1s* spectra of the nMgO/ITO film at a binding energy of 531.1 eV^25^. The observed peaks with binding energies at 528.8, 531.1, and 532.4 eV corresponds to MgO, Mg(OH)_2_, and MgCO_3_, respectively. The other peaks with binding energies at 528.8 and 531.1 eV are due to the nMgO and its oxygen vacancies, respectively. After immobilization of pDNA (Fig. 3D), a slight shift in the binding energy values occurs upon deconvolution, which indicates the changes due to DNA functionalization. Two additional peaks at 535.4 and 537.1 eV towards higher binding energies are due to the negatively charged DNA backbone electrostatically attached to the positively charged MgO NPs. Table 1 shows the relative atomic percentage (%) of different peaks observed in the nMgO/ITO and pDNA-nMgO/ITO films. After pDNA coating on the surface of nMgO, the relative atomic percentage (%) of the peak at 528.8 eV decreases to 12.4%, but the peak at 531.1 eV increases by 4.8% due to incorporation of the water molecules with pDNA. Thus, this shift of binding energies and appearance of new O*1s* peaks confirm the pDNA functionalization onto the surface of nMgO.

Figure 4(A) shows the PL emission spectra obtained for bare nMgO, pDNA immobilized nMgO, and hybridized cDNA (400 ng/μL) with pDNA on the nMgO surface. PL studies were used to confirm the DNA hybridization on the nMgO surface. A broad red emission band of nMgO was observed at 700 nm due to the oxygen ion vacancies (F and F^+^ centers). The defects or excess surface states may be created due to movement of the atoms and ions at the lattice sites. In addition, red emission of the nMgO occurs due to the relaxation of defect centers created by the mechanical stress during fracture and rapid crystallization^13^. At the excitation wavelength (260 nm), two additional weak shoulder bands at ~440 nm and 520 nm were noted due to the free excitonic recombination of F centres (oxygen vacancies)^26^. The entire PL emission spectrum was acquired in the range of 400–700 nm. A noticeable increase in PL intensity indicates interaction of DNA with the nMgO surface and formation of a DNA-nMgO complex (Fig. 4B). This increase may be due to the strong binding tendency of the negatively charged DNA molecules with positively charged nMgO through electrostatic bound to the nMgO surface and forms a pDNA-nMgO complex. It appears that the oxygen defects and various F and F^+^ centres in nMgO are responsible for the observed PL^27^, indicating that nMgO is a suitable nanoprobe for detection of the oligonucleotide hybridization. When the cDNA is present in the added sample solution, DNA hybridization occurs between the cDNA and the surface captured pDNA and displays a cDNA concentration-dependent PL intensity increase (Fig. 4C).

## Discussion

The MgO NPs were synthesized using a sol-gel (chemical co-precipitation) method and characterized by spectroscopic and microscopic techniques. The high crystallinity of the MgO NPs was confirmed by XRD, and the particle size and morphological shape of the synthesized NPs were determined using TEM studies. The 23-base pDNA was designed from a highly virulent strain of *V. cholerae* (O1 gene) and conjugated onto the nMgO surface for the fabrication of a PL-based genosensor using hybridization.

The PL response of the fabricated genosensor was measured as a function of cDNA concentration ranging from 100 to 500 ng/μL. The PL measurements were carried out at an excitation wavelength of 260 nm as a function of cDNA concentration (Fig. 4B). A gradual increase in the PL peak intensity with increasing cDNA concentration was observed at ~700 nm and can be correlated with the intercalation of pDNA and cDNA onto nMgO, which acts as a DNA detection probe via hybridization (Fig. 4B). The increase in the peak intensity with increasing cDNA concentration is found to be linear, and has a sensitivity of 1.306 emi/ng (Fig. 4C). The sensor response varies with cDNA concentration according to the equation (1):


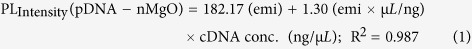


The lower detection limit (LOD) of the sensor is calculated to be as 3.133 ng/μL using the formula 3σ/m, where σ is the standard deviation (SD) and m is the slope of the curve in the linearity range i.e. 100-500 ng/μL. The sensor response varies with cDNA concentration according to the equation (2):


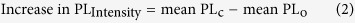


where PL_O_ is the average PL intensity for zero control (pDNA) and PL_c_ is the average PL intensity for various cDNA concentrations.

These results suggest that nMgO is an effective photoactive probe that can recognize cDNA of *V. cholerae* in the presence of pDNA. This indicates that electrostatic interactions are the major driving force for absorption of individual DNA molecules onto the nMgO surface. Tang *et al.* developed a PL-based DNA biosensor using gallium arsenide, and the enhancement of the PL signal was attributed to the passivation effect generated from the interactions of thiolated DNA and the surface of the semiconductor material^28^. It appears that in our device, the hybridization between the immobilized pDNA and cDNA provides access to the guanine bases on the surface of nMgO, which may be responsible for the observed enhanchment of PL intensity with increasing cDNA concentration.

An analytical device for cholera detection based on antibody-conjugated ZrO_2_ NPs has recently been reported^29^. In another study, an electrochemcial gold electrode modified with polytryamine was found to be sensitive at attomolar concentrations of cholera^30^. Ouerghi *et al.* used an electrodeposited film of biotinylated polypyrole to detect *V. cholerae* in the range of 10 to 80 ng/mL by DNA hybridization^31^. However, the nMgO-based PL genosensor provides improved sensitivity (1.306 emi/ng) for a wide range of cholera levels (100-500 ng/μL) compared to sensitivities reported in the literature which are listed in Table 2. The biocompatibility of the pDNA conjugated with nMgO along with the excellent PL property of nMgO is advantageous for use in an optical diagnostic biomedical device. Furthermore, this approach offers promise for the development of a commercially viable metal oxide-based genosensor for the detection of *V. cholerae* at an early stage.

The remarkable PL properties of well-dispersed, hexagonal nMgO with red emission at 700 nm in phosphate buffer solution offer good sensitivity with a wide detection range, and fast response time. Cytotoxicity of MgO NPs has also been investigated earlier using the MTT [3-(4, 5-dimethylthiazole-2-yl)-2, 5-diphenyl tatrazoliumbromide] assay in the concentration range of 50 to 350 μg/mL^32^. The results of these studies suggest that pDNA conjugated with the nMgO can be used for the development of a new generation of *in vivo* biomedical sensors, implantable biochips and for development of compact devices for detection of other virulent infectious bacterial and viral diseases such as meningitis, tuberculosis, and dengue.

## Methods

### Chemicals and reagents

Magnesium nitrate [Mg(NO_3_)_2_.6H_2_O] and oxalic acid (H_2_C_2_O_7_.2H_2_O) were procured from Merck (Mumbai, India). Sodium dihydrogen ortho-phosphate (NaH_2_PO_4_) and *di*-sodium hydrogen orthophosphate (Na_2_HPO_4_) were purchased from Qualigens Fine Chemicals Pvt., Ltd., Mumbai, India. Phosphate buffered saline (PBS, 50 mM) pH 7.0 was prepared using monobasic sodium phosphate and dibasic sodium phosphate solutions with 0.9% NaCl. DNA solutions were prepared in Tris EDTA buffer (TE, 10 mM Tris, 1 mM EDTA, pH 8.0). All solutions were prepared using deionized water (Milli Q 10 TS), and glassware was autoclaved prior to use. The single stranded capture probe DNA (pDNA) sequence 5′-GCATATGCAAATGGAACACCTCA-3′ was procured from the Midland Certified Reagent Company, Midland, Texas, USA for DNA hybridization studies.

### Sample preparation and probe design

Patient samples were collected from the National Centre for Disease Control (NCDC) in New Delhi, India, and the complementary genomic DNA of *V. cholerae* was isolated as per the standardized research protocol using organic solvents such as phenol-chloroform mixture and RNase followed by precipitation with ethanol^33^. The virulent O1 gene sequence of *V. cholerae* was identified from the National Center for Biotechnology Information (NCBI) database, and homology of the sequence was further confirmed using the Basic Local Alignment Search Tool.

### Material synthesis

A sol-gel method was used to synthesize MgO NPs. Magnesium nitrate and oxalic acid were used as precursor materials and were mixed at a 4:1 molar ratio as reported previously after slight modification^34^. pH of the solution was neutralized (~7.0) by several washings with autoclaved distilled water, after which the solution was evaporated at 80 °C followed by drying at 120 °C in a vacuum oven to dry the gel. The dried gel was further calcined at 900 °C for about 3 h to obtain nMgO powder, which was then used for characterization and functionalization of the pDNA molecules.

### Functionalization of probe DNA

**Figure 1:**
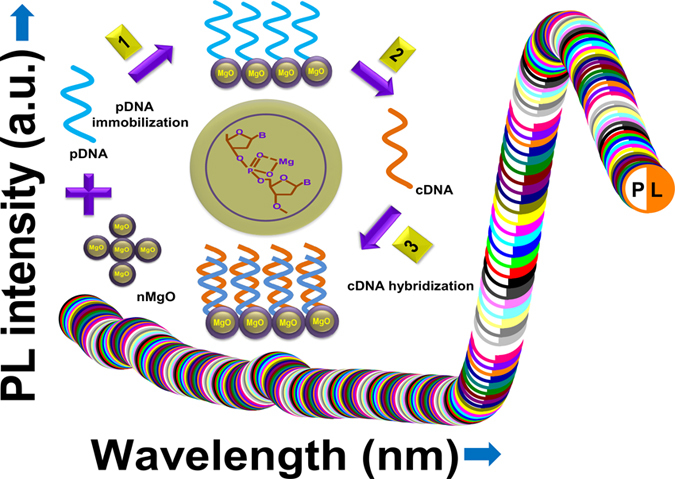
Schematic shows the fabrication steps of the label-free optical PL genosensor.

**Figure 2:**
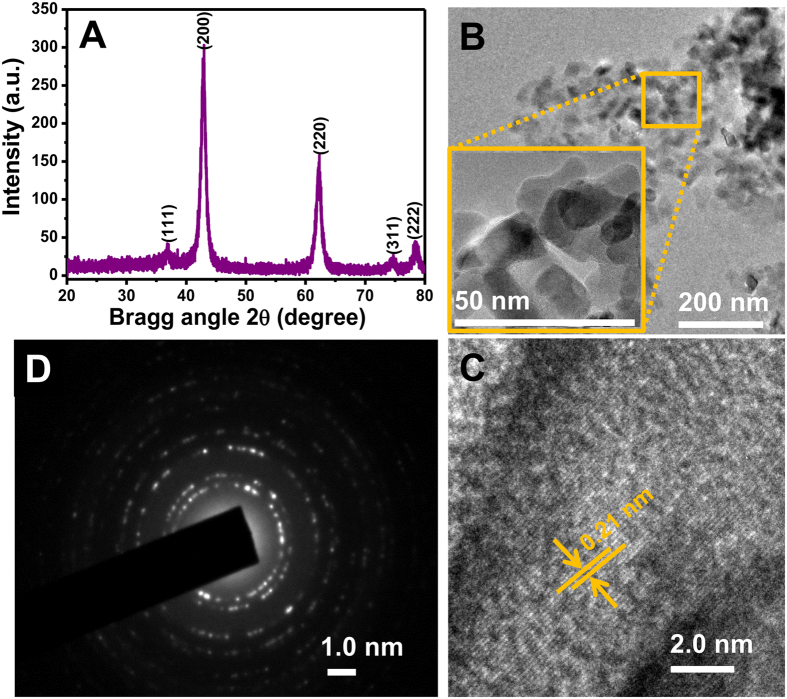
(**A**) Powder XRD pattern of MgO NPs. (**B**) TEM image of the well-distributed nMgO (inset: high magnification image of hexagonal nMgO NPs). (**C**) High resolution image for viewing lattice fringes of nMgO. (**D**) SAED pattern of nMgO.

**Figure 3:**
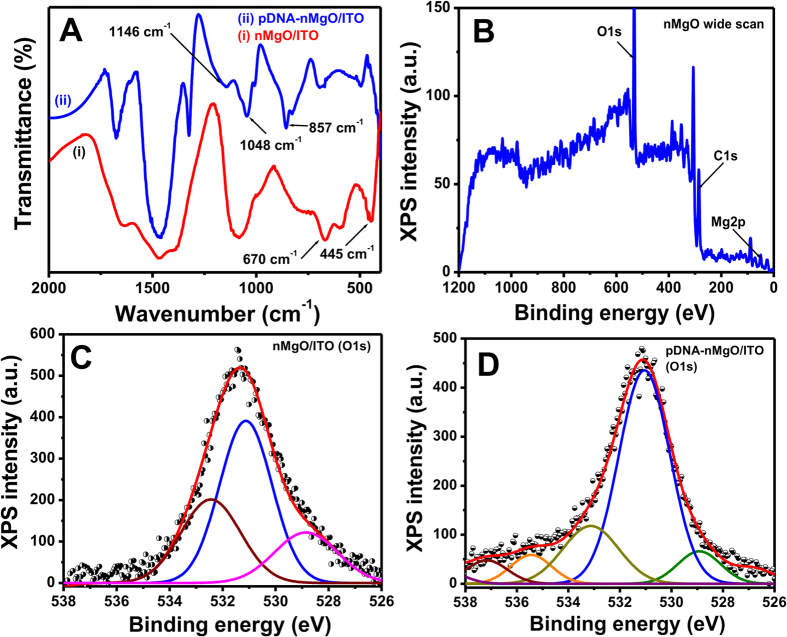
(**A**) FT-IR Spectra of bare nMgO (i) and after functionalization with pDNA (ii). (**B**) Survey scan XPS spectra of nMgO/ITO film. (**C**) O1s core level spectra of nMgO/ITO film. (**D**) O1s core level spectra of pDNA-nMgO/ITO film (The original data is shown as scatter points while the fitting data is shown by solid lines).

**Figure 4:**
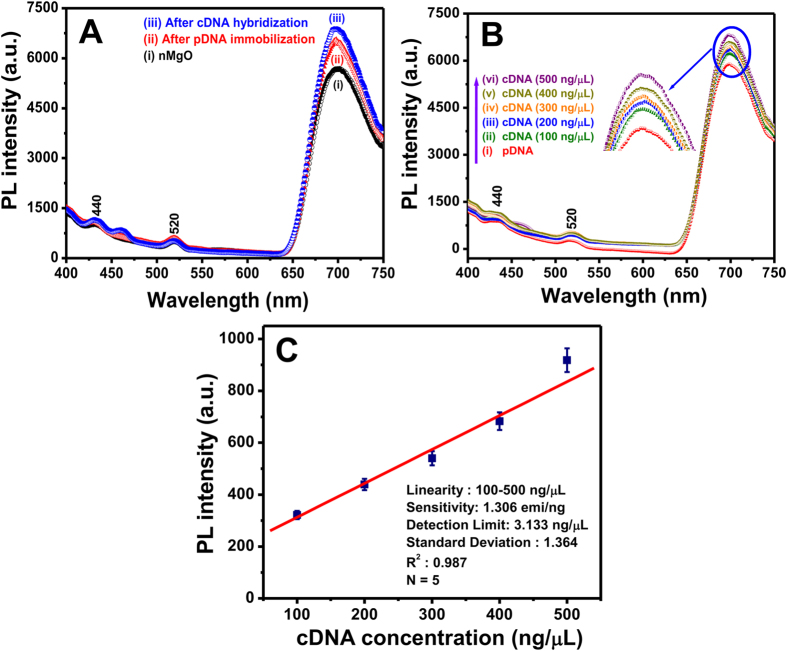
(**A**) PL spectra of (i) bare nMgO, (ii) with pDNA immobilization onto nMgO surface, and (iii) After cDNA hybridization onto pDNA-nMgO in solution phase. (**B**) PL response studies after cDNA hybridization onto nMgO surface at different concentration range (100–500 ng/μL) in PBS (50 mM, pH 7.0, containing 0.9% NaCl). (**C**) Genosensor calibration plot for detection of cDNA concentration (100–500 ng/μL) from the increase in PL intensity.

First, 1.0 mg of MgO NPs was dispersed in 1 mL of deionized water and ultra-sonicated to obtain a uniform dispersion. The capture pDNA solution (10 pM/μL) was prepared in TE buffer (pH 8.0) for immobilization onto desired nMgO surface. For DNA hybridization studies, complementary cDNA was prepared in the solution phase after ultra-sonication (24 KHz, Vibronics Pvt. Ltd., Mumbai, India) for 20 min to break longer cDNA strands into smaller fragments^35^. These cDNA fragments were later denatured at 95 °C for ~5 min to separate the DNA strands, immediately followed by ice treatment for 1 min prior to hybridization with the pDNA-conjugated nMgO. The schematic of fabrication process of the label-free optical PL genosensor is shown in Fig. 1.

### Instrumentation

Structural information about the synthesized nMgO was obtained by using X-ray diffraction spectroscopy (XRD, Rigaku) with Cu-Kα (λ = 1.542Å) X-ray source. Fourier transform infrared spectroscopic data was obtained with FT-IR, from Perkin-Elmer, Model 2000 and XPS measurements were carried out using an S-Probe ESCA Model 2803 (Fision Instrument, 10 kV, 20 mA) with AlKα as the X-ray source. The morphology of the nMgO was observed by a high resolution-transmission electron microscope (HR-TEM, JEOL- 2100F, 200 KV), and photoluminescence measurements were conducted using a Luminescence Spectrometer (Edinburg F 900).

## Additional Information

**How to cite this article**: Patel, M. K. *et al.* A Label-Free Photoluminescence Genosensor Using Nanostructured Magnesium Oxide for Cholera Detection. *Sci. Rep.* **5**, 17384; doi: 10.1038/srep17384 (2015).

## Figures and Tables

**Table 1:** The O*1s* core level spectra of nMgO/ITO and pDNA-nMgO/ITO films.

Sample details	Fitting of the O 1s peak Binding energy [eV], Relative atomic percentage (%)				
	MgO	Mg(OH)_2_	MgCO_3_		
nMgO/ITO	528.8(19.7)	531.1(52.7)	532.4(30.7)	–	–
pDNA-nMgO/ITO	528.9(7.34)	531.0(57.5)	533.1(17.0)	535.4(6.2)	537.1(5.1)

**Table 2:** Comparative study of various biosensing characteristics for *V. cholerae* detection reported in the literature.

Surface/Matrix	Transducer	Detection limit (ng/μL)	Detection range (ng/μL)	Sensitivity	References
Polytyramine-modified gold electrode	Immunosensor/Electrochemical	0.09 aM	10^−19^−10^−11^	–	30
Electrodeposited polypyrrole	Immunosensor	0.01	10–80	–	31
Microcapillary based	Immunosensor	6.6 × 10^−11^	1.0 × 10^7^–1.6 × 10^7^	–	36
Gold electrode	DNA biosensor/Electrochemical	100	100–500	0.027 μA/ng/cm^2^	37
nZrO_2_/ITO	Immunosensors/Electrochemical	10 aM	10 × 10^−8^ − 10 nM	2.34 μA/nM	29
nMgO/ITO	DNA biosensor/Electrochemical	59.12	0–500	16.80 nA/ng/cm^2^	34
nMgO-CH/ITO	DNA biosensor/Electrochemical	35.20	100–500	36.72 nA/ng/cm^2^	32
nMgO	DNA biosensor/Optical	74.39	100–500	1.434 emi/ng	[Present Work]
